# Guanylate-binding protein-5 is involved in inflammasome activation by bacterial DNA but only the cooperation of multiple GBPs accounts for control of *Brucella abortus* infection

**DOI:** 10.3389/fimmu.2024.1341464

**Published:** 2024-02-08

**Authors:** Fabio V. Marinho, Camila Brito, Ana Carolina V. S. C. de Araujo, Sergio C. Oliveira

**Affiliations:** ^1^ Instituto de Ciências Biológicas, Departamento de Bioquímica e Imunologia, Universidade Federal de Minas Gerais, Belo Horizonte, Brazil; ^2^ Instituto de Ciências Biológicas, Departamento de Genética, Ecologia e Evolução, Universidade Federal de Minas Gerais, Belo Horizonte, Brazil; ^3^ Instituto de Ciências Biomédicas, Departamento de Imunologia, Universidade de São Paulo, São Paulo, Brazil

**Keywords:** guanylate-binding proteins, GBP5, inflammasome, DNA, *Brucella abortus*

## Abstract

**Introduction:**

Guanylate-binding proteins (GBPs) are produced in response to pro-inflammatory signals, mainly interferons. The most studied cluster of GBPs in mice is on chromosome 3. It comprises the genes for GBP1-to-3, GBP5 and GBP7. In humans, all GBPs are present in a single cluster on chromosome 1. Brucella abortus is a Gram-negative bacterium known to cause brucellosis, a debilitating disease that affects both humans and animals. Our group demonstrated previously that GBPs present on murine chromosome 3 (GBPchr3) is important to disrupt Brucella-containing vacuole and GBP5 itself is important to Brucella intracellular LPS recognition. In this work, we investigated further the role of GBPs during B. abortus infection.

**Methods and results:**

We observed that all GBPs from murine chromosome 3 are significantly upregulated in response to B. abortus infection in mouse bone marrow-derived macrophages. Of note, GBP5 presents the highest expression level in all time points evaluated. However, only GBPchr3-/- cells presented increased bacterial burden compared to wild-type macrophages. Brucella DNA is an important Pathogen-Associated Molecular Pattern that could be available for inflammasome activation after BCV disruption mediated by GBPs. In this regard, we observed reduced IL-1β production in the absence of GBP2 or GBP5, as well as in GBPchr3-/- murine macrophages. Similar result was showed by THP-1 macrophages with downregulation of GBP2 and GBP5 mediated by siRNA. Furthermore, significant reduction on caspase-1 p20 levels, LDH release and Gasdermin-D conversion into its mature form (p30 N-terminal subunit) was observed only in GBPchr3-/- macrophages. In an *in vivo* perspective, we found that GBPchr3-/- mice had increased B. abortus burden and higher number of granulomas per area of liver tissue, indicating increased disease severity.

**Discussion/conclusion:**

Altogether, these results demonstrate that although GBP5 presents a high expression pattern and is involved in inflammasome activation by bacterial DNA in macrophages, the cooperation of multiple GBPs from murine chromosome 3 is necessary for full control of Brucella abortus infection.

## 1 Introduction

Interferons (IFNs) are crucial cytokines of the host’s defense against microorganisms. IFNs signaling leads to the induction of genes called IFN-stimulated genes (ISGs) that will act as potent inducers of antimicrobial effector response against intracellular bacteria. Among the most important ISGs are GTPases. Currently, four families of IFN-induced GTPases are described, namely Immunity-Related GTPases (IRGs), Myxoma resistance proteins (MX), Very Large Inducible GTPases (VLIGs/GVINs), and Guanylate-Binding Proteins (GBPs) (1, 2). GBPs range from 65 to 73 kDa and are among the most abundantly IFN-gamma induced genes (3). These proteins are included in the dynamin GTPase protein superfamily, alike the other IFN-induced GTPases (2). GBP genes are clustered on two chromosomes in mice. One cluster on chromosome 3 contains GBP1, GBP2, GBP3, GBP5 and GBP7 genes, while another cluster on chromosome 5 includes GBP4, GBP6, GBP8, GBP9, GBP10, GBP11 genes. Conversely, humans have 7 GBPs (numbered 1-to-7) and all of them are in a single cluster on chromosome 1 (4, 5). A new classification of GBPs has been proposed recently, which is based on phylogenetic analysis. According to that, only GBP2, GBP5 (both in chromosome 3), and GBP6 (chromosome 5) are orthologs from primates’ GBPs. The other GBPs genes were incorrectly annotated and the nomenclature should change (6). As this is still a matter of debate, herein we will use the former nomenclature, with murine GBPs numbered from 1-to-11. GBPs are relevant for protection against intracellular bacteria, including *Listeria monocytogenes, Mycobacterium bovis, Francisella novicida*, and *Chlamydia sp* (7–11). Moreover, these proteins also have roles in the immune responses against viruses and parasites, e.g. being related to resistant phenotype against Ectromelia virus infection and cerebral toxoplasmosis (12, 13). In cancer as well, GBPs are involved in improved response against tumor growth (14). Among GBPs antimicrobial functions, we can include induction of inflammasome activation, direct killing of the pathogen, changes in the autophagy pathway, and release of Pathogen-Associated Molecular Patterns (PAMPs) that can activate the cell (11, 15). These functions can take place concurrently, as found in *F. novicida* infection (10), or unlinked, as happens during *Chlamydia* infection, where GBPs-mediated inflammasome activation is uncoupled from vacuole rupture or direct killing (8).

*Brucella abortus* is Gram-negative bacterium classified as facultative intracellular. It infects mainly macrophages and dendritic cells, which will function as replicative niches for this pathogen. To survive and replicate inside host cells, *B. abortus* induces the formation of a membranous compartment known as *Brucella*-containing vacuole (BCV) (16). This compartment interacts with Endoplasmic Reticulum (ER) and is an important step of the *Brucella* intracellular cycle (16). Several virulence factors help this bacterium to replicate while remaining stealthy. For instance, the VirB Type IV secretion system is necessary to deliver virulence factors that will control BCV-ER interactions. Also, *Brucella* LPS is non-classical, being less toxic and less active in Toll-Like Receptor 4 (TLR4) stimulation than other Gram-negative bacteria (17). Thus, comprehension of the mechanisms that permeate this host-pathogen interaction can help to deal better with stealthy infections.

Overall, the cluster of GBPs from murine chromosome 3 (from here on referred to as GBPchr3) are the most studied, notably GBP1, GBP2 and GBP5 (18). Our group has demonstrated that GBPchr3 is important to induce BCV disruption and inflammasome activation, increasing bacterial burden upon *B. abortus* infection (19). Furthermore, GBP5 has been shown to participate in *B. abortus* LPS intracellular recognition in macrophages and non-canonical inflammasome activation (20). Another important PAMP that may gain access to host cytosol after BCV disruption is bacterial DNA. Once in the cytoplasm, it can activate STING or AIM2 inflammasome pathways (19, 21). It was shown that activation of AIM2 by *B. abortus* DNA is essential for IL-1β production and control of *B. abortus* infection (21). However, it is still unclear if GBP5 also influences on *Brucella* DNA inflammasome activation and protection against brucellosis. In this study, we show that GBP5 participates in bacterial DNA-inflammasome activation, although cooperation of multiple GBPs from murine chromosome 3 is necessary for defense against *B. abortus* infection.

## 2 Materials and methods

### 2.1 Animals

C57BL/6 wild-type mice were obtained from the Federal University of Minas Gerais (UFMG) animal facility. Dr. Thirumala-Devi Kanneganti from St. Jude Children’s Research Hospital, Memphis, USA, provided GBP5^-/-^ mice. Dr. Petr Broz from the University of Lausanne, Lausanne, Switzerland, provided GBPchr3^-/-^ and GBP2^-/-^ mice. All mice were kept in a laboratory facility free of pathogens. Male and female mice were used at 8-12 weeks of age. All experimental protocols were reviewed and approved by the Animal Studies Committee (protocol CEUA/UFMG 69/2020).

### 2.2 Bacteria, growth conditions and DNA extraction

*Brucella abortus* virulent strain S2308 from our bacteria library was grown in *Brucella* broth (BB; BD Biosciences, Franklin Lakes, NJ) for 3 days under constant agitation (180 RPM, 37°C). Bacterial suspension was pelleted (4000 RPM, 4°C), suspended in glycerol 10% and frozen at -80°C until use. Prior to *in vitro* infection or DNA extraction, freshly unfrozen *B. abortus* was grown in BB under constant agitation at 37°C. *B. abortus* DNA was obtained using Illustra bacteria genomicPrep Spin Kit (GE HealthCare, Chicago, IL) accordingly to manufacturer instructions.

### 2.3 Generation of bone marrow-derived macrophages and *in vitro* stimulation

Macrophages were obtained from the bone marrow of mice (bone marrow-derived macrophages; BMDMs), as described in (11). Briefly, bone marrow cells were removed from femurs and tibias and cultured in DMEM (Gibco/Thermo Fisher Scientific, Waltham, MA) containing 10% heat-inactivated FBS (Gibco/Thermo Fisher Scientific), 1% HEPES, penicillin G sodium (100 U/mL), streptomycin sulfate (100 μg/mL) and 20% L929 cell-conditioned medium (LCCM) in petri dishes (approximately 1 x 10^7^ cells/petri dish). Cells were incubated at 37°C with 5% CO_2_ for a total of 7 days. On the fourth day of culture, 10 mL of fresh medium was added. At seventh day, macrophages were detached and seeded in 24-well plates (5 x 10^5^ cells/well) and used for *in vitro* studies. To stimulate BMDMs, *B. abortus* (MOI 100:1) suspended in supplemented DMEM (1% FBS, 1% HEPES) was added. To evaluate inflammasome activation mediated by *B. abortus* DNA, cells were first primed with Pam3CSK4 (Pam, 1 μg/mL; InvivoGen, San Diego, CA) for 4 hours. Following, medium was changed and cells were transfected with bacterial DNA (1 μg/mL) using FuGENE HD (Promega, Madison, WI), accordingly to manufacturer instructions. Unless otherwise specified, stimulation was performed for 17 hours. As a positive control for inflammasome activation, cells were primed with Pam (1 μg/mL) for 4 hours following addition of 20 μM nigericin sodium salt (Sigma-Aldrich) in the last 45 minutes of incubation. Stimulation with Pam (1 μg/mL) for 17h was used as internal control. Culture supernatants were collected and subsequently used for cytokine analysis, western blot, or LDH release evaluation. IL-1β, TNF-α, IL-6 and IL-12p40 production was measured in the supernatants of cells by ELISA using a DuoSet kit (R&D Systems, Minneapolis, MN), according to the manufacturer’s guidelines.

### 2.4 Knockdown of GBP genes in THP-1 cells and BMDMs via small interfering RNA

THP-1 cells culture was performed as described in (22). THP-1 macrophages were then transfected with siRNA from siGENOME SMARTpools (Dharmacon, Lafayette, CO) using the GenMute siRNA transfection reagent according to the manufacturer’s instructions (SignaGen, Rockville, MD). siGENOME SMARTpool siRNAs specific for human GBP1 (M-005153-02-0005), GBP2 (M-011867-00-0005), GBP3 (M-031864-01-0005), GBP5 (M-018178-00-0005), and GBP7 (M-032072-01-0005) were used in this study. A control siRNA pool was used (D-001206-14-05). After 42 hours of siRNA transfection, cells were stimulated as described for BMDMs. Culture supernatants were collected and subsequently used for cytokine analysis and LDH release evaluation. Human IL-1β production was measured in the supernatants of cells by ELISA using a DuoSet kit (R&D Systems). Alternatively, BMDMs from C57BL/6 wild-type mice were transfected with siGENOME SMARTpool siRNAs specific for mouse GBP2 (M-040199-00-0005), GBP5 (M-054703-01-0005), or submitted to an integrated co-transfection using a pre-mixture of both siRNAs to achieve double knockdown of GBP2 and GBP5. A control siRNA pool was used. Forty-two hours later, cells were stimulated as described above for evaluation of IL-1β production and LDH release.

### 2.5 Quantitative real-time PCR

BMDMs were stimulated in 24-well plates and homogenized in TRIzol reagent (Invitrogen, Carlsbad, CA, USA) to obtain total RNA accordingly to manufacturer guidelines. Quantitative Real-Time PCR was performed as described in (11). Briefly, cDNA was synthesized by reverse transcription of 2 µg of total RNA. The Quantitative Real-Time PCR reaction used SYBR Green PCR Master Mix (Thermo Fischer Scientific) and was performed in a QuantStudio 3 Real-Time PCR System (Thermo Fischer Scientific). Primers used to amplify a specific fragment corresponding to the gene target are shown in **Table 1**. Data analysis was performed using the threshold cycle (ΔΔC_t_) method and results were presented as relative expression units after normalization to the housekeeping gene. PCR measurements were conducted in duplicates.

**Table 1:** Primers for quantitative real-time PCR.

Primers	Forward 5’-3’	Reverse 5’-3’
mGBP1	GAGTACTCTCTGGAAATGGCCTCAGAAA	TAGATGAAGGTGCTGCTGAGGAGGACTG
mGBP2	CTGCACTATGTGACGGAGCTA	CGGAATCGTCTACCCCACTC
mGBP3	CTGACAGTAAATCTGGAAGCCAT	CCGTCCTGCAAGACGATTCA
mGBP5	CTGAACTCAGATTTTGTGCAGGA	CATCGACATAAGTCAGCACCAG
mGBP7	TCCTGTGTGCCTAGTGGAAAA	CAAGCGGTTCATCAAGTAGGAT
mβ-actin	GGCTGTATTCCCCTCCATCG	CCAGTTGGTAACAATGCCATGT

### 2.6 Western blot

Western blot for evaluating inflammasome activation was performed as described previously (23). Briefly, cell lysate from BMDM cultures was harvested after *in vitro* stimulation. Cell lysis was performed with Mammalian Protein Extraction Reagent (M-PER) (Thermo Fisher Scientific) supplemented with 1 mM Na_3_VO_4_, 10 mM NaF and 1:100 of protease inhibitor cocktail (Sigma-Aldrich). Equal protein amounts (25 μg of cell lysate) or equal supernatant volumes (20 μL) were subjected to electrophoresis on 15% polyacrylamide gel, followed by western blotting according to standard techniques. Anti-mouse primary antibodies were used in this study: Mouse monoclonal caspase-1 (p20) (clone Casper 1; AdipoGen Life Sciences, San Diego, CA, USA), Rabbit monoclonal GSDMD (clone EPR19828; Abcam, Cambridge, UK) and Rabbit monoclonal β-actin (clone 12E5; Cell Signaling Technology, Danvers, MA, USA). Loading control (β-actin) was performed using primary antibodies at a 1:5000 dilution. For detection of the other targets, the primary antibodies were used at 1:1000 dilution. Immunoreactive bands were visualized using Luminol chemiluminescent HRP substrate (Millipore, Billerica, MA, USA) in an Amersham Imager 600 (GE HealthCare). Intensity of bands was quantified and normalized using ImageJ software (National Institutes of Health, Bethesda, MD, USA; available at http://imagej.nih.gov/ij/). Unless otherwise specified, numbers below each band represent its intensity relative to the respective β-actin.

### 2.7 Quantification of lactate dehydrogenase release

The activity of lactate dehydrogenase enzyme was evaluated using a CytoTox96 LDH release kit (Promega) according to the manufacturer’s instructions.

### 2.8 *In vitro* infection and measurement of bacterial intracellular growth

BMDMs (5 × 10^5^ cells; 24-well plates) were plated with DMEM supplemented with 1% FBS and 1% HEPES. Cells were infected with *B. abortus* (MOI 100:1) in 300 μL/well of medium and were incubated for 2 hours at 37°C in a 5% CO_2_ atmosphere. Next, to remove noninternalized bacteria, the cells were washed with warm saline and incubated in DMEM supplemented with 10% FBS and 1% HEPES for 24 hours (37°C; 5% CO_2_). To obtain the number of intracellular bacteria, macrophages were lysed 2 h (T0) and 24 hours post-infection with sterile and ice-cold H_2_O. Serial dilutions were plated in BB agar medium, and the colony-forming units (CFUs) were counted after 3 days of incubation at 37°C.

### 2.9 *In vivo* infection and immune evaluation

Mice were challenged intraperitoneally with 1 x 10^6^ CFU *B. abortus*. Two weeks post-infection, mice were euthanized and both spleen and liver were harvested aseptically from each animal. Spleens were homogenized in saline (NaCl 0.9%), serially diluted, and plated on BB agar for measurement of bacterial burden. The plates were incubated at 37°C for 3 days for CFU determination. The results are shown as Log_10_ CFU mean per gram of organ. Alternatively, spleen cells (1 × 10^6^ cells; 96-well plates) were plated with RPMI supplemented with 10% FBS and penicillin G sodium (100 U/mL)/streptomycin sulfate (100 μg/mL). Cells were infected with *B. abortus* (MOI 100:1). Stimulation with *E. coli* LPS (1 μg/mL) or Concanavalin A (ConA; 5 μg/mL) were used as internal controls. Cells were incubated for 48 hours or 72 hours at 37°C in a 5% CO_2_ atmosphere for measurement of TNF-α and IFN-γ levels, respectively, in the supernatants by ELISA using a DuoSet kit (R&D Systems).

### 2.10 Histopathology

The liver histopathology was performed as described before (11). Briefly, the liver of infected mice was collected and immediately fixed in a 10% buffered formaldehyde solution. Next, tissue was dehydrated, diaphanized, and embedded in paraffin. Tissue sections (2–3 𝜇m) were stained with Hematoxylin and Eosin (H&E). Digital images of the slides were obtained with an Olympus SC30 camera (Olympus, Tokyo, Japan) using 10× objective lens. Granulomas were identified and quantified by observing the presence of round-shaped cellular infiltrates in the tissue parenchyma. Using ImageJ software, the total area of ​​each section was quantified by converting pixels to square millimeters. Results are expressed as number of granulomas per square centimeter of tissue.

### 2.11 Statistics

Results are shown as the mean ± SD. Statistically significant differences between the groups were evaluated by two-way ANOVA followed by the Bonferroni *post hoc* test (*p* < 0.05) or one-way ANOVA followed by the Tukey *post hoc* test (*p* < 0.05). GraphPad Prism 9.0 (GraphPad Software, San Diego, CA, USA) was used for the analyses.

## 3 Results

### 3.1 GBP5 is upregulated in response to *B. abortus* but cooperation of multiple GBPs is required for control of bacterial growth in macrophages

**Figure 1:**
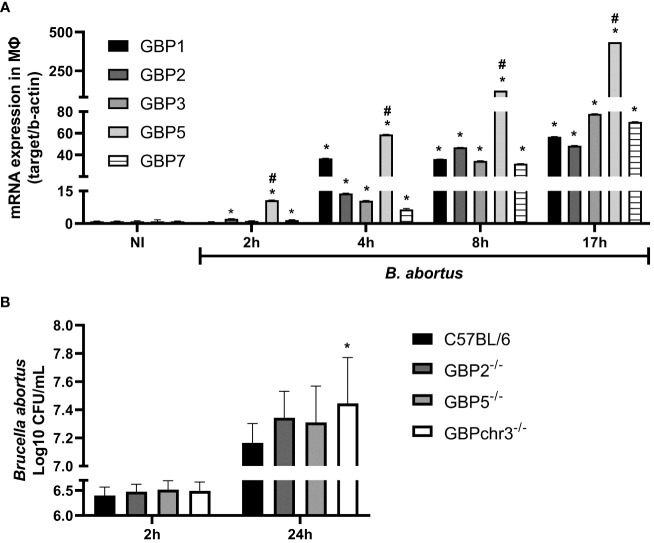
GBP5 presents fast expression profile but the participation of multiple GBPs is required to control *B*. *abortus* intracellular growth in macrophages. **(A)** BMDMs were obtained from C57BL/6 mice and infected with *B*. *abortus* (MOI 100:1). The expression kinetics of GBPs from murine chromosome 3 was assessed up to 17 hours post-infection. *Significant compared to NI; #Significant compared to GBP1, GBP2, GBP3 and GBP7. Data shown are representative of three independent experiments performed. **(B)** BMDMs from wild-type, GBP2^-/-^, GBP5^-/-^ or GBPchr3^-/-^ C57BL/6 mice were infected with *B*. *abortus* (MOI 100:1). After 2 hours cells were washed to remove noninternalized bacteria. The number of intracellular *B*. *abortus* was obtained by lysis of macrophages at 2 hours and 24 hours post-infection. *Significant compared to C57BL/6. Data shown are pooled from three independent experiments performed.

The GBPs are strongly responsive to IFN-γ but are also induced in response to type-I IFN, TNF-α, IL1-β, IL-1α and TLR agonists (5). However, *Brucella* has different mechanisms to promote a stealthy infection (17). First, we determined the kinetics of GBPs expression in *B. abortus* infected wild-type BMDMs. As early as 2 hours post-infection, we observed significantly increased GBP2, GBP5 and GBP7 mRNA levels compared to non-infected cells (**Figure 1A**). However, only after 4 hours of infection all GBPs evaluated reached significantly higher expression compared to non-infected cells. These higher expression levels of GBPs remained up to 17 hours, the last time point investigated. Interestingly, GBP5 showed the highest expression level in all time points studied among the GBPs evaluated. Therefore, for the following experiments we selected GBP5 to investigate its role during *B. abortus* infection. Besides GBP5, we also studied GBP2, due to its high expression profile and its aggregation near BCVs during infection (19). Next, we investigated whether higher expression of GBP2 and GBP5 would influence bacterial burden in macrophages. BMDMs from GBP2^-/-^ or GBP5^-/-^ single deficient mice, from GBPchr3^-/-^ (deficient in all GBPs from murine chromosome 3) and from wild-type mice, were compared concerning *B. abortus* intracellular growth control. Despite the absence of GBP2 or GBP5 did not increase bacterial burden in macrophages (p=0.22 GBP2^-/-^ versus wild-type; p=0.47 GBP5^-/-^ versus wild-type), GBPchr3^-/-^ cells presented significant replication of intracellular *B. abortus* compared to wild-type cells (**Figure 1B**). Noteworthy, bacterial burden in GBP2^-/-^ and GBP5^-/-^ macrophages are not statistically different from GBPchr3^-/-^ cells, despite being smaller (p=0.77 GBP2^-/-^ versus GBPchr3^-/-^; p=0.52 GBP5^-/-^ versus GBPchr3^-/-^). This finding indicates that deficiency on GBP2 or GBP5 alone slightly hampers the control of *B. abortus* growth, but not to a level where bacterial burden becomes significantly higher than wild-type macrophages, like in GBPchr3^-/-^ cells. Together, these data suggest although GBP2 and GBP5 showed high and fast expression pattern, remarkably GBP5, the cooperation of GBPs is necessary for control of *B. abortus* growth intracellularly.

### 3.2 GBP2 and GBP5 are important for inflammasome activation mediated by *B. abortus* DNA

**Figure 2:**
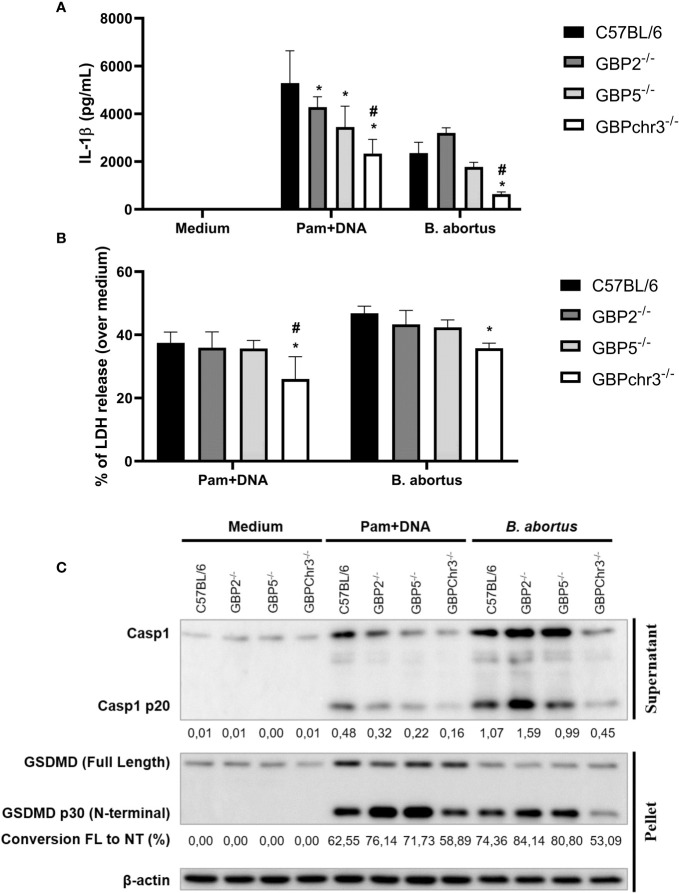
GBP2 and GBP5 contribute to *B*. *abortus* DNA-triggering of inflammasome response, but full activation requires GBPchr3 in macrophages. BMDMs from wild-type, GBP2^-/-^, GBP5^-/-^ or GBPchr3^-/-^ C57BL/6 mice were infected with *B*. *abortus* (MOI 100:1) for 17 hours. Alternatively, BMDMs were primed with Pam3CSK4 (Pam; 1 μg/mL) for 4 hours. Following, the medium was changed and cells were transfected with *B*. *abortus* DNA (1 μg/mL) to evaluate inflammasome activation (17 hours of DNA stimulation). The levels of **(A)** IL-1β production and **(B)** LDH release were assessed in supernatants. *Significant compared to wild-type C57BL/6 BMDMs; #Significant compared to GBP2^-/-^ and GBP5^-/-^ BMDMs. **(C)** p20 subunit of caspase-1 was measured in supernatants while GSDMD was evaluated in cell’s lysate. For caspase-1 p20, the quantification of densitometry is expressed as target relative to the housekeeping protein β-actin. For GSDMD, the conversion of Full Length (FL) protein into p30 N-terminal fragment (NT; mature form) was quantified by densitometry. Data shown are representative of three independent experiments performed.

**Figure 3:**
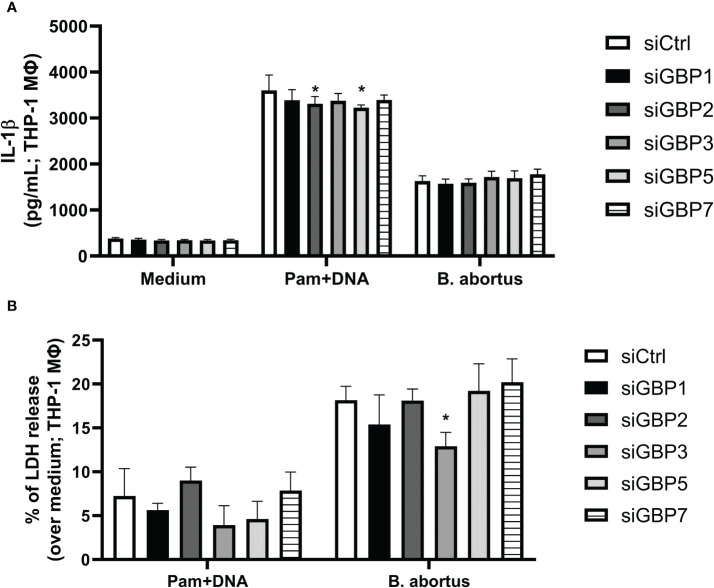
GBP2 and GBP5 contribute to IL-1β production in *B. abortus* DNA-stimulated THP-1 cells. THP-1 cells were differentiated into macrophages in the presence of PMA (100 nM; 48 hours). THP-1 macrophages were then transfected with siRNA for knocking down human GBPs (siGBPs) or control siRNA pool (siCTL). Forty-two hours after siRNA transfection, cells were infected with *B*. *abortus* (MOI 100:1) for 17 hours. Alternatively, macrophages were primed with Pam3CSK4 (Pam; 1 μg/mL) for 4 hours. Following, the medium was changed and cells were transfected with *B*. *abortus* DNA (1 μg/mL) to evaluate inflammasome activation (17 hours of DNA stimulation). The levels of **(A)** IL-1β production and **(B)** LDH release were assessed in supernatants. *Significant compared to siCTL. Data shown are representative of three independent experiments performed.

During the *B. abortus* intracellular cycle, bacterial DNA may become available in cytosol to activate inflammasome pathway after BCV disruption (19). That said, we investigated if GBP2 or GBP5 plays a role in *B. abortus* DNA recognition in macrophages. To mimic this scenario, cells were primed with Pam3CSK4 (Pam) for 4 hours followed by transfection of *B. abortus* DNA to evaluate inflammasome activation. In our experimental design, Pam (TLR2 ligand) was used as first signal replacing LPS (TLR4 ligand) to avoid type-I IFN production, which could cause induction on GBPs expression and mask the results. As shown in **Figure 2**, we found GBP2^-/-^ and GBP5^-/-^ BMDMs presented reduced IL-1β production and caspase-1 cleavage into its mature form (p20 subunit). Interestingly, GBPchr3^-/-^ BMDMs showed further reduction in IL-1β and caspase-1 p20 levels, and diminished LDH release and Gasdermin-D (GSDMD) conversion into its mature form (p30 N-terminal subunit). These data suggest GBP2 and GBP5 are important for mature IL-1β secretion but all GBPs from murine chromosome 3 act synergically for full inflammasome activation mediated by bacterial DNA. In a different manner, deficiency in GBP2 or GBP5 alone does not compromise inflammasome activation induced by *B. abortus* infection. In this context, only GBPchr3^-/-^ BMDMs presented diminished IL-1β, caspase-1 p20 and GSDMD p30 levels, and reduced LDH release (**Figures 2A–C**). To evaluate whether these findings could be extended to human macrophages, THP-1 monocyte lineage was used. We knocked down with siRNA all orthologs of murine GBPchr3 and reproduced the conditions of infection or *B. abortus* DNA inflammasome activation as in murine macrophages. The silencing of single human GBPs (hGBPs) expression was not sufficient to alter IL-1β production upon *B. abortus* infection (**Figure 3A**). However, downregulation of hGBP2 and hGBP5 expression resulted in small but significant reduction in IL-1β levels in response to bacterial DNA, like what was found with BMDMs (**Figure 3A**). Additionally, knockdown of hGBP2 or hGBP5 did not change significantly LDH release (**Figure 3B**). These data corroborate the findings in mouse macrophages. Moreover, deficiency in GBPs in BMDMs or knockdown of hGBPs in THP-1 cells did not change inflammasome activation upon Pam + Nigericin stimulation, a NLRP3 inflammasome inducer, indicating that downregulation of GBPs does not influence inflammasome induction by this agonist (**Supplementary Figure 1**). To further address the interplay of GBPs, we knocked down simultaneously GBP2 and GBP5 in BMDMs. Interestingly, double knockdown of GBP2 and GBP5 results in additional reduction on Pam+DNA-induced IL-1β production compared to single knockdown of either GBP2 or GBP5 (**Supplementary Figure 2**). Moreover, reduced LDH release was also found in GBP2/5 double silenced BMDMs, although it did not reach statistical significance (p=0.06 comparing siGBP2/5 versus siCTL; **Supplementary Figure 2**). These data suggest that GBP2 and GBP5 may cooperate to mediate DNA-induced inflammasome activation. It is important to mention that production of other proinflammatory cytokines, namely TNF-α, IL-6 and IL-12, by BMDMs in response to *B. abortus* infection was not influenced by deficiency of GBPs (**Supplementary Figure 3**). These results indicate that GBP2 and GBP5 have a role in inflammasome activation mediated by intracellular bacterial DNA in both murine and human macrophages, but multiple GBPs are required for full inflammasome activation upon infection.

### 3.3 Cooperation of multiple GBPs is necessary to control *B. abortus* infection *in vivo*

**Figure 4:**
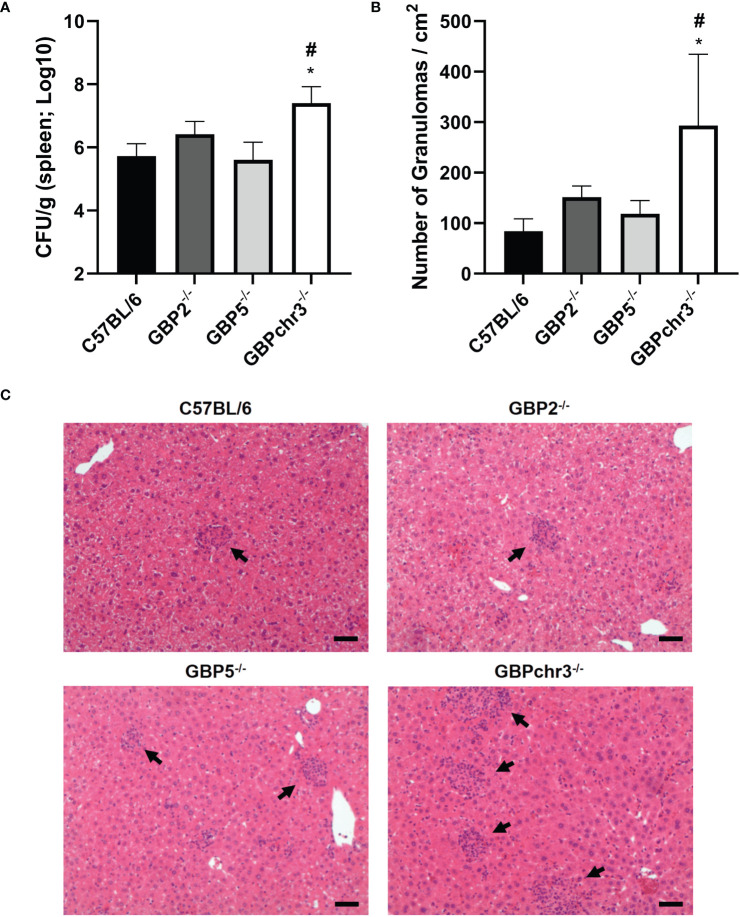
Multiple GBPs from murine chromosome 3 are required for *in vivo* control of *B*. *abortus* infection. Wild-type, GBP2^-/-^, GBP5^-/-^ and GBPchr3^-/-^ C57BL/6 mice were infected i.p. with 1x10^6^ *B*. *abortus* and monitored for two weeks post-infection. **(A)** The spleen of infected mice was individually macerated, serially diluted, and plated for colony-forming units (CFU) counting. Bacterial burden is shown as Log_10_ CFU mean per gram of organ. *Significant compared to wild-type C57BL/6 mice; #Significant compared to GBP2^-/-^ and GBP5^-/-^ mice. **(B)** The liver of infected mice was collected, embedded in paraffin, and stained with H&E. Slides were evaluated for the number of granulomas and normalized by the covered area (in square centimeters). *Significant compared to wild-type C57BL/6 mice. **(C)** Representative images of each group are shown. The arrows indicate examples of granulomas. Scale bars: 50µm. Data shown are representative of three independent experiments performed.

Brucellosis is a debilitating infection that tends to become chronic (24). The innate immune responses triggered in early phase of the disease can influence its progression. Since GBP5 is highly induced in macrophages upon *B. abortus* infection, affecting bacterial DNA inflammasome activation, we wonder if this GBP would influence *in vivo* protection. In this regard, C57BL/6 wild-type, GBP2^-/-^, GBP5^-/-^ and GBPchr3^-/-^ mice were infected with *B. abortus* and monitored for two weeks post-infection (**Figure 4**). The single deficiency in GBP5 or GBP2 did not change either the bacterial burden assessed in spleen or the number of granulomas per area found in liver, compared to wild-type mice. Conversely, deficiency in the cluster of GBPs from murine chromosome 3 resulted in increased susceptibility to infection, with enhanced *B. abortus* burden and number of granulomas in mouse livers. Noteworthy, the production of TNF-α and IFN-γ by spleen cells recall response against re-stimulation with *B. abortus* was not influenced by deficiency of GBPs (**Supplementary Figure 3**). These data suggest multiple GBPs cooperation is necessary for *in vivo* control of bacterial growth and correlated granuloma formation. Altogether, these results indicate GBP5 has the fastest and highest expression profile among the GBPs present in mouse chromosome 3 upon *B. abortus* infection, influencing bacterial DNA inflammasome activation. However, the cooperation of these GBPs is required for host protection.

## 4 Discussion

Brucellosis is a widespread zoonosis that affects a great diversity of hosts. The investigation of immune mechanisms that could influence this disease progression is relevant since low antibiotic treatment success due to high relapse rate of infection is very common in humans (25). In addition, there is no vaccine available to be used on human beings (25). In this study, we found that all GBPs contained in the murine chromosome 3 (GBP1-3, 5 and 7) are significantly upregulated during *B. abortus* infection in BMDMs. Of note, GBP5 presents the fastest and highest expression level. Additionally, both GBP5 and GBP2 influence inflammasome activation mediated by *Brucella* DNA, once deficiency in either GBPs leads to reduced caspase-1 p20 cleavage and IL-1β production. Likewise, the knockdown of GBP5 and GBP2 by siRNA in THP-1 macrophages resulted in decreased IL-1β secretion levels. Nevertheless, severe impairment of *B. abortus*-elicited immune response in macrophages was observed only due to the knockout of all GBPs from murine chromosome 3. This condition rendered cells with decreased ability to control bacterial intracellular growth, hampered inflammasome activation, represented by diminished IL-1β, caspase-1 p20 and GSDMD p30 levels, and reduced LDH release. Additionally, a similar response was observed when GBPch3^-/-^ macrophages were stimulated with *Brucella* DNA delivery in cytosol. These findings correlated with the *in vivo* investigation, where we found that only GBPchr3^-/-^ mice had increased disease severity. Thus, GBP5 and GBP2 contribute to inflammasome activation mediated by intracellular bacterial DNA, but multiple GBPs cooperation is necessary for host protection against *B. abortus* infection.

IFN-induced GTPases are important for an adequate antimicrobial response, particularly against vacuolar pathogens (2). GBPs are conserved between mouse and human and can be triggered by a diverse set of inflammatory signals. They participate in cell-autonomous immune response against various intracellular pathogens. GBPs main functions involve the disruption of parasite-containing vacuoles or microbial membranes, and the supporting for assemble inflammasome complexes and/or recruitment of antimicrobial effectors (26). We found GBPs 1-3, 5 and 7 reach significantly increased expression after 4 hours following *B. abortus* infection. Remarkably, GBP5 presents increased induction 2 hours post-infection and it remains with the highest expression level in all time points evaluated (up to 17 hours). Indeed, as early as 6 hours post-infection with *B. abortus*, it is possible to observe BCV rupture in macrophages, a process that is significantly reduced in GBPchr3^-/-^ cells (19). Moreover, correlating with our data, GBP5 is found among the most expressed genes, presenting several orders of magnitude over non-stimulated cells in *F. novicida* infected BMDMs and LPS + IFN-γ induced M1 human macrophages (10, 27).

The release of bacterial products by GBP-mediated lysis of pathogen’s vacuoles or cells activates cytosolic immune pathways, including inflammasomes (26). Here in, we show that GBPchr3*^-/-^* macrophages present impaired inflammasome activation and GSDMD cleavage upon *B. abortus* infection. This observation supports previous findings by our group where GBPchr3 deficient BMDMs were unable to trigger pore formation in response to infection, a process that was shown to be dependent on GSDMD and caspase-11 (20). *Brucella* LPS and GBP5 were identified as important players in this context (20). Another PAMP that becomes available after GBPs-mediated disruption of membranes (of vacuoles or the pathogen itself) is bacterial DNA. It was shown that during *F. novicida* infection, GBPs and AIM2 act ensemble to provide a DNA-based inflammasome activation (9, 10). In this regard, we showed both GBP5 and GBP2 working in *B. abortus* DNA-dependent inflammasome activation. THP-1 macrophages presented similar phenotype, expanding this observation to human cells. It is worth mentioning that GBP5 and GBP2 participated in IL-1β production and caspase-1 activation, but did not influence GSDMD conversion or LDH release. Thus, other GBPs may collaborate in resolving downstream steps of inflammasome activation and pyroptotic fate decision. In accordance, diminished DNA-mediated inflammasome activation was observed in GBPchr3^-/-^ BMDMs, being significantly lower than levels found in GBP5^-/-^ or GBP2^-/-^ cells. Overall, this scenario suggests that several GBPs cooperate in the DNA-mediated inflammasome activation, which was not observed for LPS triggering of the noncanonical inflammasome, where GBP5 alone played a prominent role in the process (20). Interestingly, GBPs relevance on proinflammatory cytokines production in response to *B. abortus* infection seems to be mainly concerning IL-1β production, as TNF-α, IL-6, IL-12 and IFN-γ levels remained similar among wild-type and knockout cells.

Most of the research into the mechanism of GBPs role in infections is carried out in myeloid cells, which are the main niche for *B. abortus* growth. They act as vectors for systemic dissemination to other organs, including spleen and liver, where the bacteria persist within granulomatous inflammatory lesions (24, 25). The single deficiency in GBP5 or GBP2 did not alter significantly the capacity of macrophages to control *B. abortus* replication. However, the absence of the entire cluster of GBPs on mouse chromosome 3 resulted in reduced ability of cells in limiting bacterial growth. This phenotype was translated into increased bacterial burden and elevated number of granulomas per liver tissue area in infected mice, characterizing higher severity of the disease in GBPchr3^-/-^ animals. The augmented *in vivo* susceptibility may result of a deficient recruitment and function of phagocytes, such as macrophages and neutrophils. *Brucella abortus* load often correlates with granuloma formation, which might function as reservoir of bacteria and result in chronic disease (24, 28–30). Similar results were obtained concerning other bacteria such as *M. bovis* BCG and *F. novicida*, where macrophage bacterial burden correlated with host severity of infection (7, 9–11). Indeed, in most scenarios where GBPs influence inflammasome activation, it results in increased susceptibility *in vivo*. Notwithstanding, GBPs also impact other pathways, such as autophagy induction, NADPH oxidase recruitment, or cell activation (7, 11, 31). However, while individual GBPs were identified as crucial for protection against other pathogens (7, 9, 15, 32), this was not observed in *B. abortus* infection. So far, it is the cooperation of multiple GBPs that protects the host.

**Figure 5:**
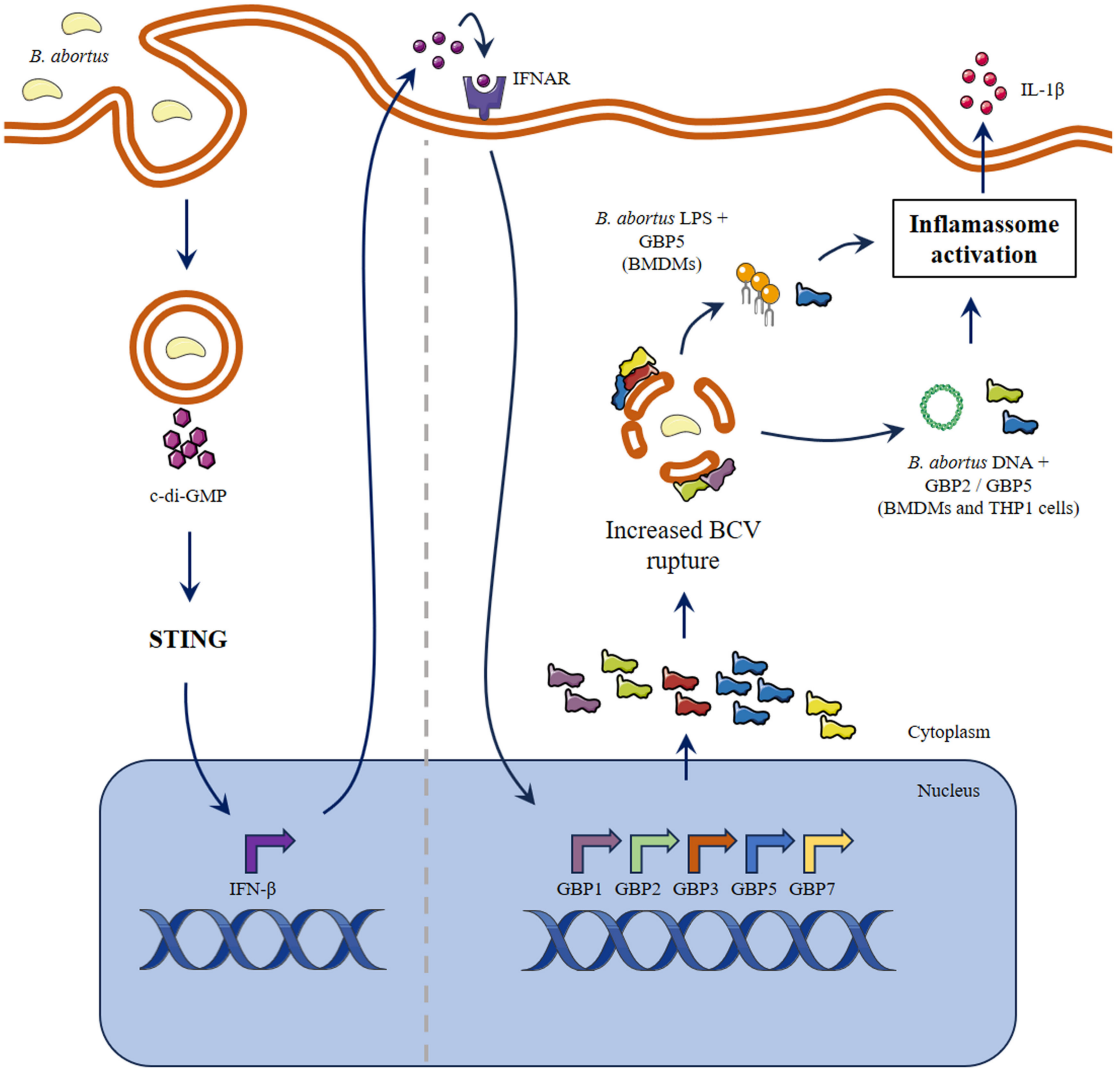
Schematic model proposed for the role of GBPs in inflammasome activation during *B*. *abortus* infection. The early interaction between macrophage and *B*. *abortus* results in STING activation mediated by bacterial cyclic dinucleotides. In response to type-I IFN and/or other pro-inflammatory stimuli, there is augmentation in expression of the cluster of GBPs on murine chromosome 3. Next, GBPs will be responsible for increased BCV disruption and PAMPs release. The induction of non-canonical inflammasome activation by LPS is dependent on GBP5. On the other hand, *B*. *abortus* DNA can activate inflammasome in a process where GBP5 and GBP2 participate. Parts of the figure were drawn by using pictures from Servier Medical Art. Servier Medical Art by Servier is licensed under a Creative Commons Attribution 3.0 Unported License (https://creativecommons.org/licenses/by/3.0/).

The manipulation of endosomes to become vacuoles permissive to replication or the escape to the cell cytosol are strategies employed by pathogens to limit immune stimulation. GBPs function as hubs to several intracellular pathways by disrupting parasitic niches, by assessing directly the infectious agent, and/or by acting as platforms that bridge the foreign insult and effector molecules (7, 10, 19, 33). Both murine and human GBPs form homo- and hetero-polymers to accomplish their antimicrobial function (2). In this regard, GBPs can accumulate in the pathogen’s vacuole as densely packed multimers (34). Of note, GBP1, GBP2 and GBP5 can be isoprenylated on their C-terminal domain, which confers affinity to membranes (35). It is suggested that mobilization of GBPs occurs in a hierarchical manner, with GBPs 1, 2 and 5 being the first to be recruited, followed by engagement of other GBPs and/or additional host factors (2). These characteristics place them of central importance to control intracellular infections. Nevertheless, there is emerging evidence that highly virulent pathogens can overcome GBPs activation. For example, although GBPs are important to control *M. bovis* BCG infection, this is not the case for *M. tuberculosis*. The resistance to GBPs mechanisms is, at least partially, dependent on ESX-1 secretion system that is present in *M. tuberculosis* (36). Moreover, the strain *F. tularensis* SCHU S4 (highly virulent), is not susceptible to GBP-mediated control of infection, contrasting the low virulent *F. novicida*. The findings suggest that the virulent bacterium avoids activation of this mechanism (37). In this regard, it remains to be shown if the *Brucella* VirB secretion system has any role in limiting GBPs activation or if more virulent species, such as *B. melitensis*, can subvert GBPs activation (21, 38). In summary, *B. abortus* induces a fast expression of the cluster of GBPs present on murine chromosome 3, especially GBP5. All these GBPs are important for inflammasome activation, with GBP5 or GBP2 individually playing an important role concerning the intracellular DNA-mediated induction. However, the cooperation of multiple GBPs is indispensable for limiting *Brucella* growth in macrophages and *in vivo*, contributing to less severe disease. Together with former data obtained by our group, we can set an overview of GBPs role during *B. abortus* infection. At the earliest interaction between macrophage and pathogen, there is induction of an immune response dependent on microRNAs (specifically mmu-miR-21a-5p) that will result in increased expression of GBP5 (39). This initial GBPs availability may be sufficient for incipient BCV rupture (40). Following, STING activation mediated by bacterial cyclic dinucleotides will be responsible for further augmentation in GBPs expression, leading to increased BCV disruption and release of PAMPs, including LPS and DNA (19). In this regard, GBP5 will act on LPS-induced noncanonical inflammasome activation (20). *Brucella* DNA will be free to activate AIM2 inflammasome, in a process where GBP5 and GBP2 participate, or it can also further stimulate STING in a cGAS-dependent manner (**Figure 5**) (19, 41). This infection also induces other immune responses that indirectly upregulate GBPs expression, such as Unfolded Protein Response and Galectin-3 immune activation (42, 43). The immune environment provided is responsible for limiting bacterial growth, which will reflect in fewer granulomas in affected tissues and less severe disease. Altogether, this work extends the knowledge of the relevance of GBPs on *B. abortus* infection.

## Data availability statement

The raw data supporting the conclusions of this article will be made available by the authors, without undue reservation.

## Ethics statement

Ethical approval was not required for the studies on humans in accordance with the local legislation and institutional requirements because only commercially available established cell lines were used. The animal study was approved by Animal Studies Committee (protocol CEUA/UFMG 69/2020). The study was conducted in accordance with the local legislation and institutional requirements.

## Author contributions

FM: Conceptualization, Data curation, Formal Analysis, Investigation, Writing – original draft, Writing – review & editing. CB: Conceptualization, Data curation, Formal Analysis, Investigation, Writing – review & editing. AA: Data curation, Formal Analysis, Investigation, Writing – review & editing. SO: Conceptualization, Funding acquisition, Supervision, Writing – review & editing.
