# Supplementary material for: Guanylate-binding protein-5 is involved in inflammasome activation by bacterial DNA but only the cooperation of multiple GBPs accounts for control of Brucella abortus infection
Source: Front Immunol. 2024 Feb 8;15:1341464. doi: 10.3389/fimmu.2024.1341464 (PMC10885698; doi:10.3389/fimmu.2024.1341464)
Supplement: Supplementary file 1 [file Image_1.pdf]

Supplementary Material

Fig S1

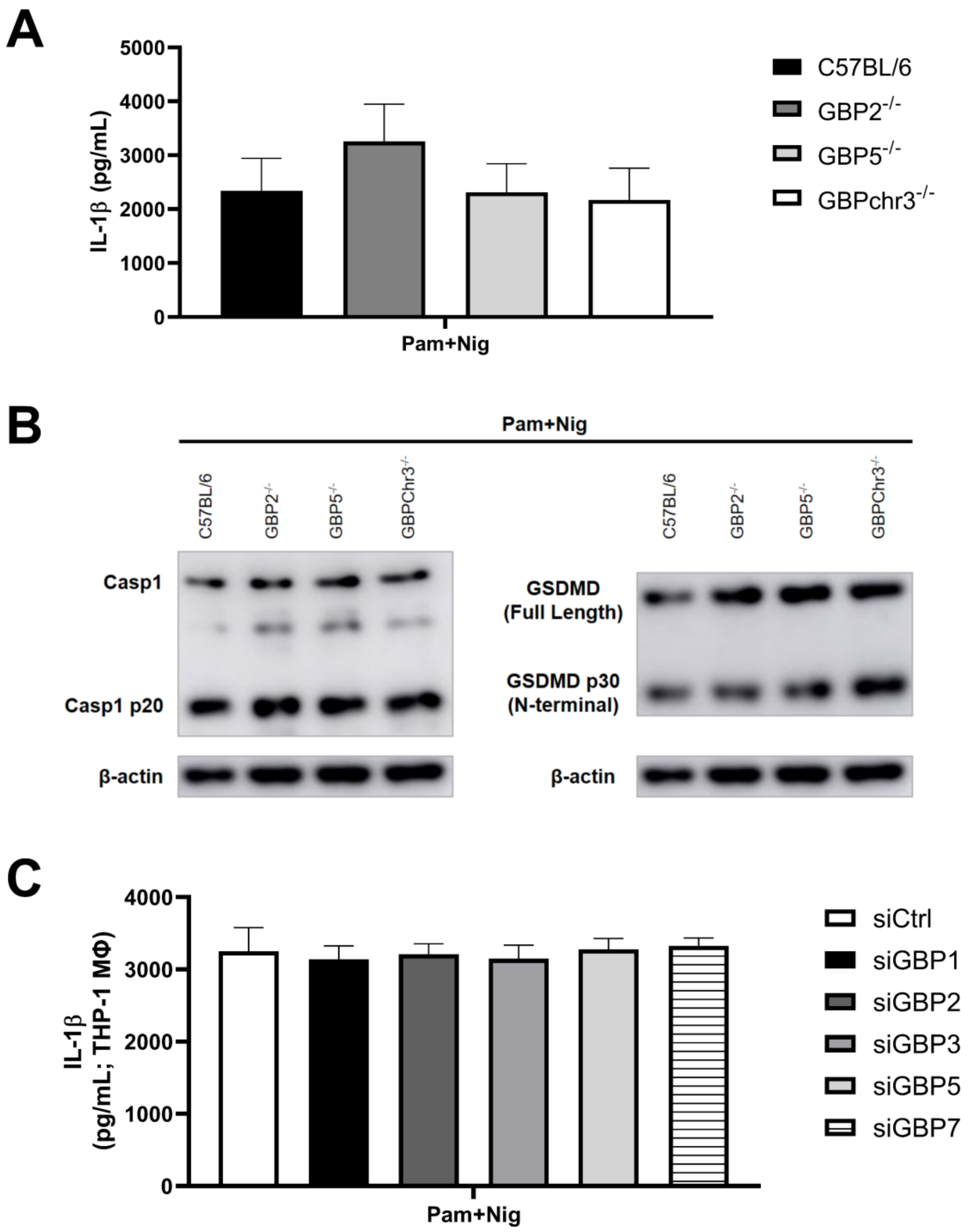

Supplementary Figure 1: Internal controls of inflammasome activation. BMDMs from wild-type, GBP2$^{-/-}$, GBP5$^{-/-}$ or GBPchr3$^{-/-}$ C57BL/6 mice were primed with Pam3CSK4 (Pam; 1 μg/mL) during 4 hours. In the last 45 min, 20 μM nigericin sodium salt (Nig) was added. **(A)** IL-1β production evaluated by ELISA. **(B)** p20 subunit of caspase-1 was measured in supernatants while GSDMD was evaluated in cell's lysate. Data shown are representative of three independent experiments performed. **(C)** THP-1 cells were differentiated into macrophages in the presence of PMA (100 nM; 48 h) and then transfected with siRNA for knocking down human GBPs (siGBPs) or control siRNA pool (siCTL). Forty-two hours after siRNA transfection, cells stimulated as described for BMDMs. The levels of IL-1β production were evaluated by ELISA. Data shown are representative of three independent experiments performed.

Fig S2

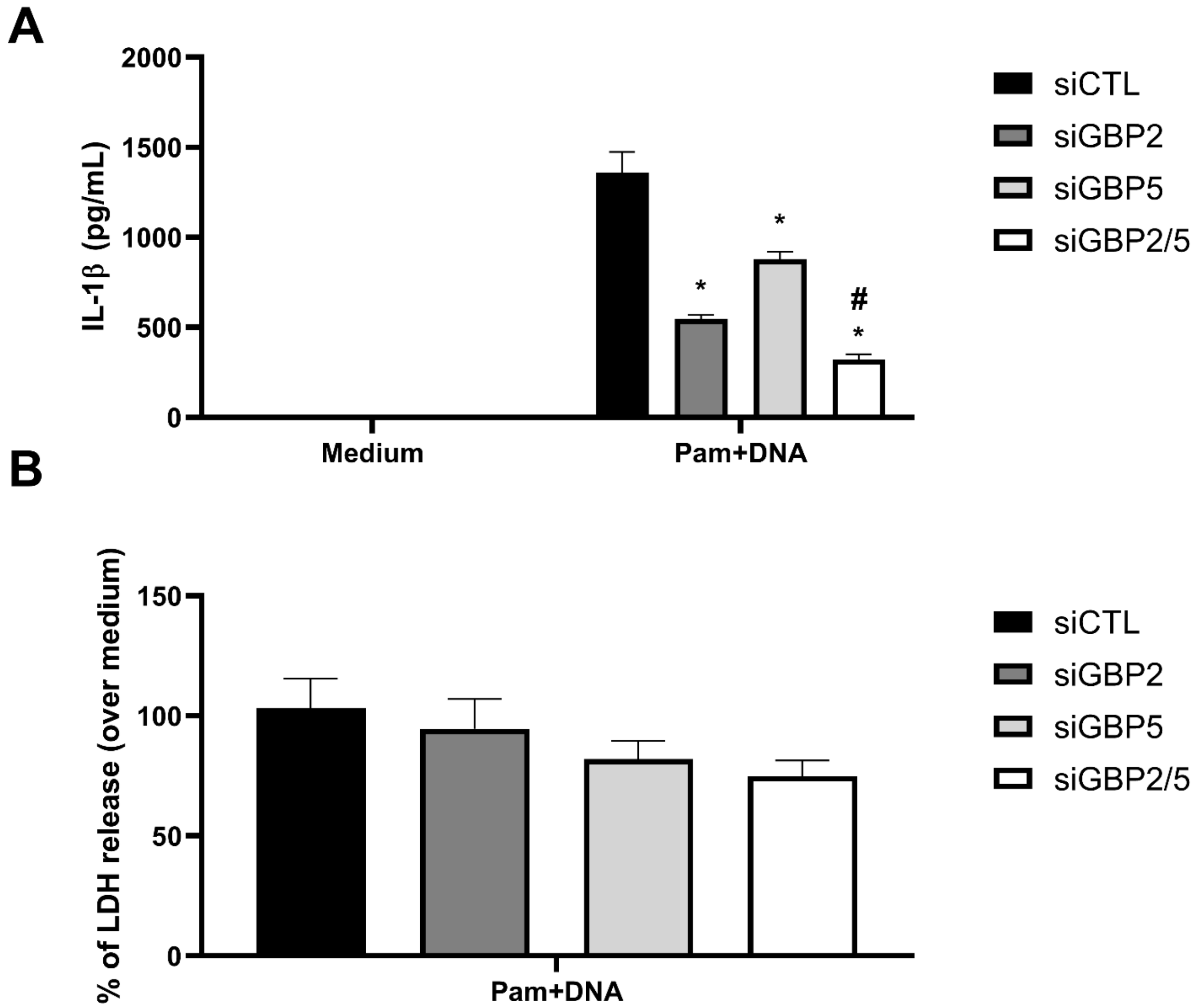

Supplementary Figure 2: Double knockdown of GBP2 and GBP5 leads to further reduction on DNA-induced IL1-β production by macrophages. BMDMs from wild-type C57BL/6 mice were transfected with siRNA for knocking down mouse GBPs (siGBPs) or control siRNA pool (siCTL). For the double knockdown of GBP2 and GBP5, cells were submitted to an integrated co-transfection using a pre-mixture of both siRNAs. Forty-two hours later, BMDMs were primed with Pam3CSK4 (Pam; 1 μg/mL) for 4 hours. Following, the medium was changed and cells were transfected with *B. abortus* DNA (1 μg/mL) to evaluate inflammasome activation (17 hours of DNA stimulation). The levels of **(A)** IL-1β production and **(B)** LDH release were assessed in supernatants. *Significant compared to siCTL; #Significant compared to siGBP2 and siGBP5. The reduction on LDH release in GBP2/5 double silenced BMDMs did not reach statistical significance (p=0.06 comparing siGBP2/5 versus siCTL).

Fig S3

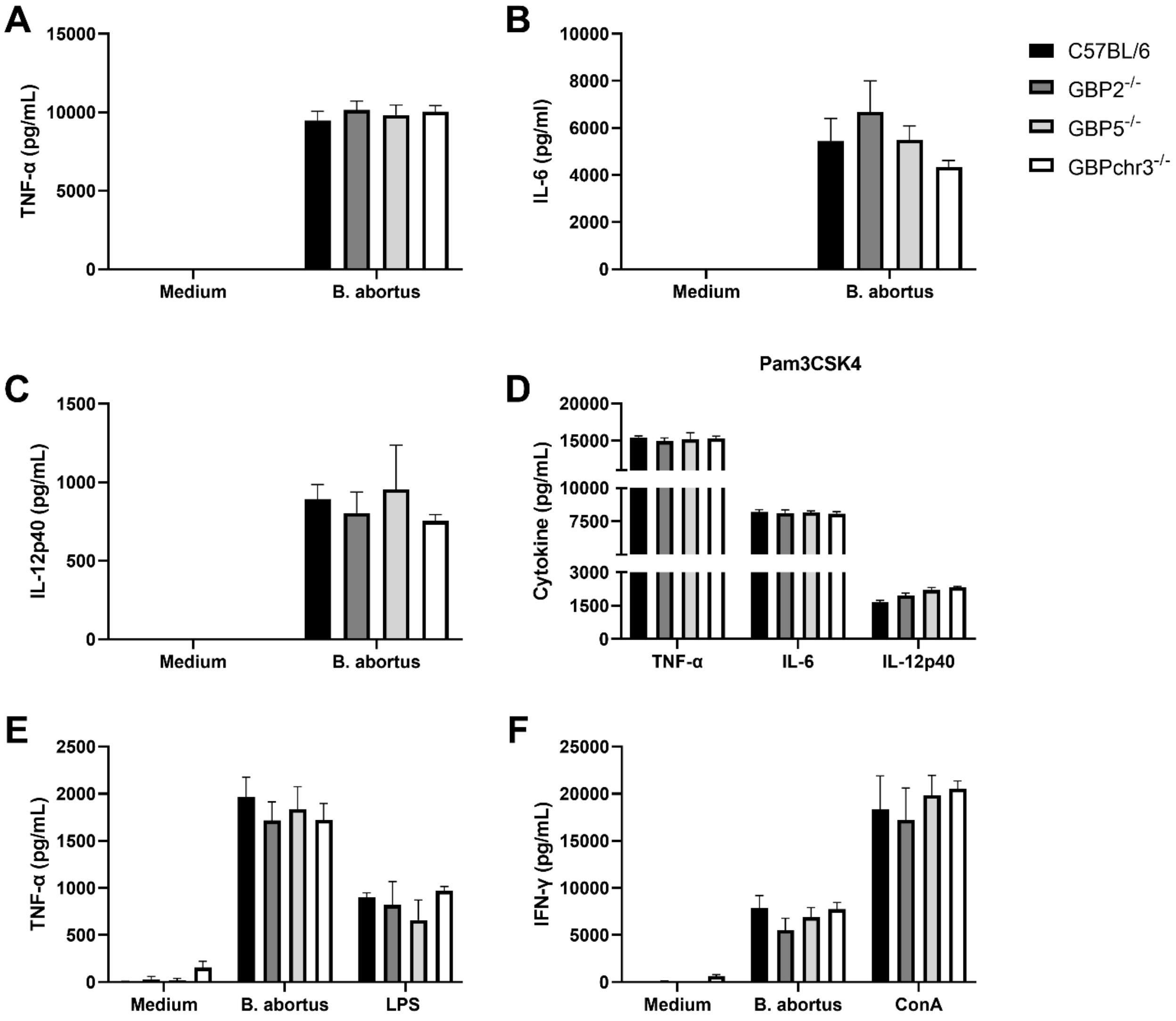

Supplementary Figure 3: GBPs from murine chromosome 3 do not influence on production of TNF-α, IL-6, IL-12 and IFN-γ during *B. abortus* infection. BMDMs from wild-type, GBP2$^{-/-}$, GBP5$^{-/-}$ or GBPchr3$^{-/-}$ C57BL/6 mice were infected with *B. abortus* (MOI 100:1) or stimulated with Pam3CSK4 (Pam; 1 μg/mL) for 17 hours. **(A-D)** The production of TNF-α, IL-6 and IL-12p40 was evaluated by ELISA. Wild-type, GBP2$^{-/-}$, GBP5$^{-/-}$ and GBPchr3$^{-/-}$ C57BL/6 mice were infected i.p. with 1x10^{6} *B. abortus* and monitored for two weeks post-infection. To evaluate the recall response, the spleen of infected mice was individually macerated, and spleen cells were stimulated with *B. abortus*. LPS and Concanavalin A (ConA) were used as internal controls for TNF-α and IFN-γ respectively. **(E-F)** The production of TNF-α and IFN-γ was evaluated by ELISA. Data shown are representative of at least three independent experiments performed.
